# Methods for the thematic synthesis of qualitative research in systematic reviews

**DOI:** 10.1186/1471-2288-8-45

**Published:** 2008-07-10

**Authors:** James Thomas, Angela Harden

**Affiliations:** 1 EPPI-Centre, Social Science Research Unit, Institute of Education, University of London, UK

## Abstract

**Background:**

There is a growing recognition of the value of synthesising qualitative research in the evidence base in order to facilitate effective and appropriate health care. In response to this, methods for undertaking these syntheses are currently being developed. Thematic analysis is a method that is often used to analyse data in primary qualitative research. This paper reports on the use of this type of analysis in systematic reviews to bring together and integrate the findings of multiple qualitative studies.

**Methods:**

We describe thematic synthesis, outline several steps for its conduct and illustrate the process and outcome of this approach using a completed review of health promotion research. Thematic synthesis has three stages: the coding of text 'line-by-line'; the development of 'descriptive themes'; and the generation of 'analytical themes'. While the development of descriptive themes remains 'close' to the primary studies, the analytical themes represent a stage of interpretation whereby the reviewers 'go beyond' the primary studies and generate new interpretive constructs, explanations or hypotheses. The use of computer software can facilitate this method of synthesis; detailed guidance is given on how this can be achieved.

**Results:**

We used thematic synthesis to combine the studies of children's views and identified key themes to explore in the intervention studies. Most interventions were based in school and often combined learning about health benefits with 'hands-on' experience. The studies of children's views suggested that fruit and vegetables should be treated in different ways, and that messages should not focus on health warnings. Interventions that were in line with these suggestions tended to be more effective. Thematic synthesis enabled us to stay 'close' to the results of the primary studies, synthesising them in a transparent way, and facilitating the explicit production of new concepts and hypotheses.

**Conclusion:**

We compare thematic synthesis to other methods for the synthesis of qualitative research, discussing issues of context and rigour. Thematic synthesis is presented as a tried and tested method that preserves an explicit and transparent link between conclusions and the text of primary studies; as such it preserves principles that have traditionally been important to systematic reviewing.

## Background

The systematic review is an important technology for the evidence-informed policy and practice movement, which aims to bring research closer to decision-making [1,2]. This type of review uses rigorous and explicit methods to bring together the results of primary research in order to provide reliable answers to particular questions [3-6]. The picture that is presented aims to be distorted neither by biases in the review process nor by biases in the primary research which the review contains [7-10]. Systematic review methods are well-developed for certain types of research, such as randomised controlled trials (RCTs). Methods for reviewing qualitative research in a systematic way are still emerging, and there is much ongoing development and debate [11-14].

In this paper we present one approach to the synthesis of findings of qualitative research, which we have called 'thematic synthesis'. We have developed and applied these methods within several systematic reviews that address questions about people's perspectives and experiences [15-18]. The context for this methodological development is a programme of work in health promotion and public health (HP & PH), mostly funded by the English Department of Health, at the EPPI-Centre, in the Social Science Research Unit at the Institute of Education, University of London in the UK. Early systematic reviews at the EPPI-Centre addressed the question 'what works?' and contained research testing the effects of interventions. However, policy makers and other review users also posed questions about intervention need, appropriateness and acceptability, and factors influencing intervention implementation. To address these questions, our reviews began to include a wider range of research, including research often described as 'qualitative'. We began to focus, in particular, on research that aimed to understand the health issue in question from the experiences and point of view of the groups of people targeted by HP&PH interventions (We use the term 'qualitative' research cautiously because it encompasses a multitude of research methods at the same time as an assumed range of epistemological positions. In practice it is often difficult to classify research as being either 'qualitative' or 'quantitative' as much research contains aspects of both [19-22]. Because the term is in common use, however, we will employ it in this paper).

When we started the work for our first series of reviews which included qualitative research in 1999 [23-26], there was very little published material that described methods for synthesising this type of research. We therefore experimented with a variety of techniques borrowed from standard systematic review methods and methods for analysing primary qualitative research [15]. In later reviews, we were able to refine these methods and began to apply thematic analysis in a more explicit way. The methods for thematic synthesis described in this paper have so far been used explicitly in three systematic reviews [16-18].

### The review used as an example in this paper

To illustrate the steps involved in a thematic synthesis we draw on a review of the barriers to, and facilitators of, healthy eating amongst children aged four to 10 years old [17]. The review was commissioned by the Department of Health, England to inform policy about how to encourage children to eat healthily in the light of recent surveys highlighting that British children are eating less than half the recommended five portions of fruit and vegetables per day. While we focus on the aspects of the review that relate to qualitative studies, the review was broader than this and combined answering traditional questions of effectiveness, through reviewing controlled trials, with questions relating to children's views of healthy eating, which were answered using qualitative studies. The qualitative studies were synthesised using 'thematic synthesis' – the subject of this paper. We compared the effectiveness of interventions which appeared to be in line with recommendations from the thematic synthesis with those that did not. This enabled us to see whether the understandings we had gained from the children's views helped us to explain differences in the effectiveness of different interventions: the thematic synthesis had enabled us to generate hypotheses which could be tested against the findings of the quantitative studies – hypotheses that we could not have generated without the thematic synthesis. The methods of this part of the review are published in Thomas *et al*. [27] and are discussed further in Harden and Thomas [21].

### Qualitative research and systematic reviews

The act of seeking to synthesise qualitative research means stepping into more complex and contested territory than is the case when only RCTs are included in a review. First, methods are much less developed in this area, with fewer completed reviews available from which to learn, and second, the whole enterprise of synthesising qualitative research is itself hotly debated. Qualitative research, it is often proposed, is not generalisable and is specific to a particular context, time and group of participants. Thus, in bringing such research together, reviewers are open to the charge that they de-contextualise findings and wrongly assume that these are commensurable [11,13]. These are serious concerns which it is not the purpose of this paper to contest. We note, however, that a strong case has been made for qualitative research to be valued for the potential it has to inform policy and practice [11,28-30]. In our experience, users of reviews are interested in the answers that only qualitative research can provide, but are not able to handle the deluge of data that would result if they tried to locate, read and interpret all the relevant research themselves. Thus, if we acknowledge the unique importance of qualitative research, we need also to recognise that methods are required to bring its findings together for a wide audience – at the same time as preserving and respecting its essential context and complexity.

The earliest published work that we know of that deals with methods for synthesising qualitative research was written in 1988 by Noblit and Hare [31]. This book describes the way that ethnographic research might be synthesised, but the method has been shown to be applicable to qualitative research beyond ethnography [32,11]. As well as meta-ethnography, other methods have been developed more recently, including 'meta-study' [33], 'critical interpretive synthesis' [34] and 'metasynthesis' [13].

Many of the newer methods being developed have much in common with meta-ethnography, as originally described by Noblit and Hare, and often state explicitly that they are drawing on this work. In essence, this method involves identifying key concepts from studies and translating them into one another. The term 'translating' in this context refers to the process of taking concepts from one study and recognising the same concepts in another study, though they may not be expressed using identical words. Explanations or theories associated with these concepts are also extracted and a 'line of argument' may be developed, pulling corroborating concepts together and, crucially, going beyond the content of the original studies (though 'refutational' concepts might not be amenable to this process). Some have claimed that this notion of 'going beyond' the primary studies is a critical component of synthesis, and is what distinguishes it from the types of summaries of findings that typify traditional literature reviews [e.g. [32], p209]. In the words of Margarete Sandelowski, *"metasyntheses are integrations that are more than the sum of parts, in that they offer novel interpretations of findings. These interpretations will not be found in any one research report but, rather, are inferences derived from taking all of the reports in a sample as a whole" *[[14], p1358].

Thematic analysis has been identified as one of a range of potential methods for research synthesis alongside meta-ethnography and 'metasynthesis', though precisely what the method involves is unclear, and there are few examples of it being used for synthesising research [35]. We have adopted the term 'thematic synthesis', as we translated methods for the analysis of primary research – often termed 'thematic' – for use in systematic reviews [36-38]. As Boyatzis [[36], p4] has observed, thematic analysis is *"not another qualitative method but a process that can be used with most, if not all, qualitative methods..."*. Our approach concurs with this conceptualisation of thematic analysis, since the method we employed draws on other established methods but uses techniques commonly described as 'thematic analysis' in order to formalise the identification and development of themes.

We now move to a description of the methods we used in our example systematic review. While this paper has the traditional structure for reporting the results of a research project, the detailed methods (e.g. precise terms we used for searching) and results are available online. This paper identifies the particular issues that relate especially to reviewing qualitative research systematically and then to describing the activity of thematic synthesis in detail.

## Methods

### Searching

When searching for studies for inclusion in a 'traditional' statistical meta-analysis, the aim of searching is to locate all relevant studies. Failing to do this can undermine the statistical models that underpin the analysis and bias the results. However, Doyle [[39], p326] states that, *"like meta-analysis, meta-ethnography utilizes multiple empirical studies but, unlike meta-analysis, the sample is purposive rather than exhaustive because the purpose is interpretive explanation and not prediction"*. This suggests that it may not be necessary to locate every available study because, for example, the results of a conceptual synthesis will not change if ten rather than five studies contain the same concept, but will depend on the range of concepts found in the studies, their context, and whether they are in agreement or not. Thus, principles such as aiming for 'conceptual saturation' might be more appropriate when planning a search strategy for qualitative research, although it is not yet clear how these principles can be applied in practice. Similarly, other principles from primary qualitative research methods may also be 'borrowed' such as deliberately seeking studies which might act as negative cases, aiming for maximum variability and, in essence, designing the resulting set of studies to be heterogeneous, in some ways, instead of achieving the homogeneity that is often the aim in statistical meta-analyses.

However you look, qualitative research is difficult to find [40-42]. In our review, it was not possible to rely on simple electronic searches of databases. We needed to search extensively in 'grey' literature, ask authors of relevant papers if they knew of more studies, and look especially for book chapters, and we spent a lot of effort screening titles and abstracts by hand and looking through journals manually. In this sense, while we were not driven by the statistical imperative of locating every relevant study, when it actually came down to searching, we found that there was very little difference in the methods we had to use to find qualitative studies compared to the methods we use when searching for studies for inclusion in a meta-analysis.

### Quality assessment

Assessing the quality of qualitative research has attracted much debate and there is little consensus regarding how quality should be assessed, who should assess quality, and, indeed, whether quality can or should be assessed in relation to 'qualitative' research at all [43,22,45]. We take the view that the quality of qualitative research should be assessed to avoid drawing unreliable conclusions. However, since there is little empirical evidence on which to base decisions for excluding studies based on quality assessment, we took the approach in this review to use 'sensitivity analyses' (described below) to assess the possible impact of study quality on the review's findings.

In our example review we assessed our studies according to 12 criteria, which were derived from existing sets of criteria proposed for assessing the quality of qualitative research [46-49], principles of good practice for conducting social research with children [50], and whether studies employed appropriate methods for addressing our review questions. The 12 criteria covered three main quality issues. Five related to the quality of the *reporting *of a study's aims, context, rationale, methods and findings (e.g. was there an adequate description of the sample used and the methods for how the sample was selected and recruited?). A further four criteria related to the sufficiency of the *strategies *employed to establish the reliability and validity of data collection tools and methods of analysis, and hence the validity of the findings. The final three criteria related to the assessment of the *appropriateness *of the study methods for ensuring that findings about the barriers to, and facilitators of, healthy eating were rooted in children's own perspectives (e.g. were data collection methods appropriate for helping children to express their views?).

### Extracting data from studies

One issue which is difficult to deal with when synthesising 'qualitative' studies is 'what counts as data' or 'findings'? This problem is easily addressed when a statistical meta-analysis is being conducted: the numeric results of RCTs – for example, the mean difference in outcome between the intervention and control – are taken from published reports and are entered into the software package being used to calculate the pooled effect size [3,51].

Deciding what to abstract from the published report of a 'qualitative' study is much more difficult. Campbell *et al*. [11] extracted what they called the 'key concepts' from the qualitative studies they found about patients' experiences of diabetes and diabetes care. However, finding the key concepts in 'qualitative' research is not always straightforward either. As Sandelowski and Barroso [52] discovered, identifying the findings in qualitative research can be complicated by varied reporting styles or the misrepresentation of data as findings (as for example when data are used to 'let participants speak for themselves'). Sandelowski and Barroso [53] have argued that the findings of qualitative (and, indeed, all empirical) research are distinct from the data upon which they are based, the methods used to derive them, externally sourced data, and researchers' conclusions and implications.

In our example review, while it was relatively easy to identify 'data' in the studies – usually in the form of quotations from the children themselves – it was often difficult to identify key concepts or succinct summaries of findings, especially for studies that had undertaken relatively simple analyses and had not gone much further than describing and summarising what the children had said. To resolve this problem we took study findings to be all of the text labelled as 'results' or 'findings' in study reports – though we also found 'findings' in the abstracts which were not always reported in the same way in the text. Study reports ranged in size from a few pages to full final project reports. We entered all the results of the studies verbatim into QSR's NVivo software for qualitative data analysis. Where we had the documents in electronic form this process was straightforward even for large amounts of text. When electronic versions were not available, the results sections were either re-typed or scanned in using a flat-bed or pen scanner. (We have since adapted our own reviewing system, 'EPPI-Reviewer' [54], to handle this type of synthesis and the screenshots below show this software.)

### Detailed methods for thematic synthesis

The synthesis took the form of three stages which overlapped to some degree: the free line-by-line coding of the findings of primary studies; the organisation of these 'free codes' into related areas to construct 'descriptive' themes; and the development of 'analytical' themes.

#### Stages one and two: coding text and developing descriptive themes

In our children and healthy eating review, we originally planned to extract and synthesise study findings according to our review questions regarding the barriers to, and facilitators of, healthy eating amongst children. It soon became apparent, however, that few study findings addressed these questions directly and it appeared that we were in danger of ending up with an empty synthesis. We were also concerned about imposing the a priori framework implied by our review questions onto study findings without allowing for the possibility that a different or modified framework may be a better fit. We therefore temporarily put our review questions to one side and started from the study findings themselves to conduct an thematic analysis.

**Figure 1:**
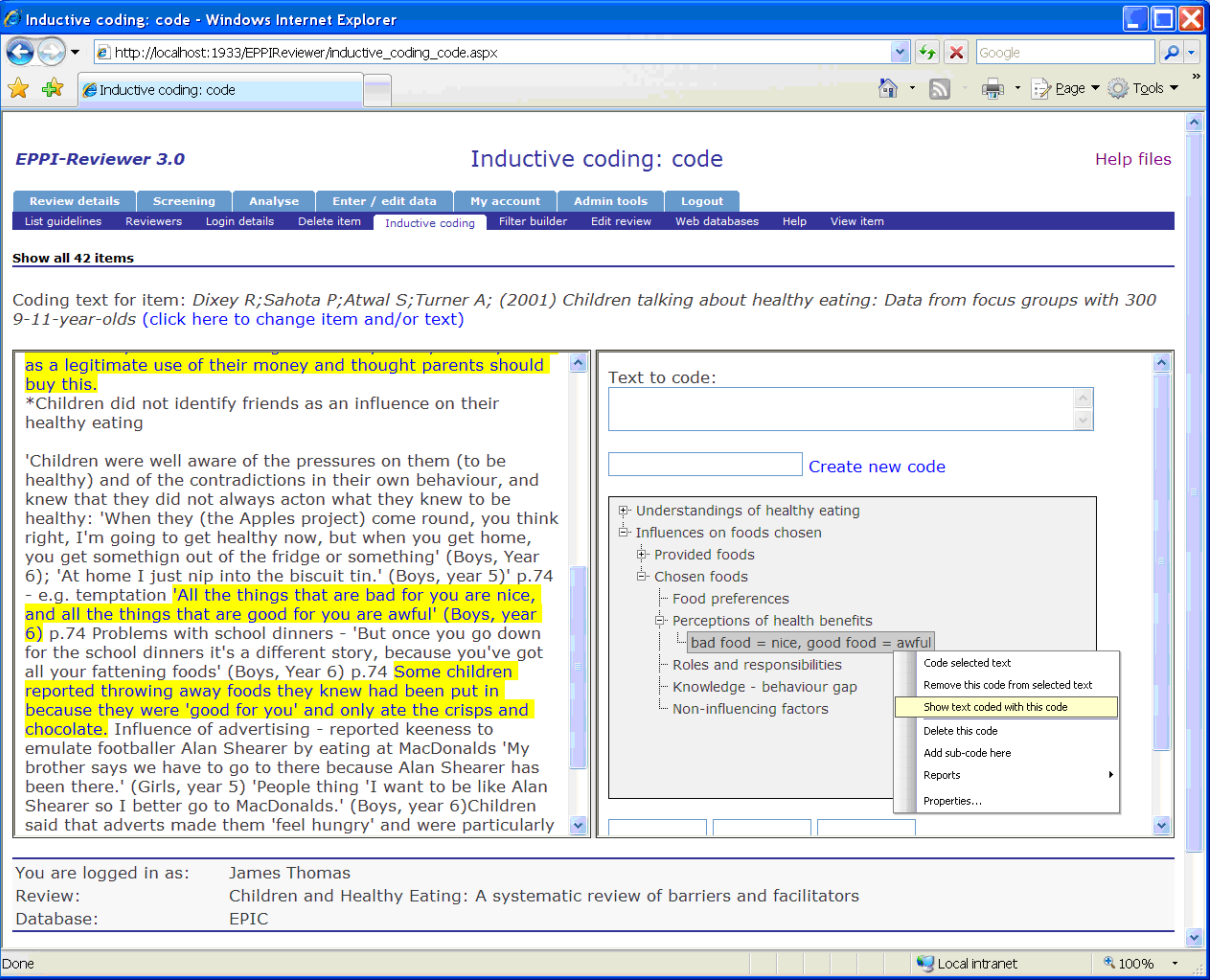
line-by-line coding in EPPI-Reviewer.

There were eight relevant qualitative studies examining children's views of healthy eating. We entered the verbatim findings of these studies into our database. Three reviewers then independently coded each line of text according to its meaning and content. Figure 1 illustrates this line-by-line coding using our specialist reviewing software, EPPI-Reviewer, which includes a component designed to support thematic synthesis. The text which was taken from the report of the primary study is on the left and codes were created inductively to capture the meaning and content of each sentence. Codes could be structured, either in a tree form (as shown in the figure) or as 'free' codes – without a hierarchical structure.

The use of line-by-line coding enabled us to undertake what has been described as one of the key tasks in the synthesis of qualitative research: the *translation *of concepts from one study to another [32,55]. However, this process may not be regarded as a simple one of translation. As we coded each new study we added to our 'bank' of codes and developed new ones when necessary. As well as translating concepts between studies, we had already begun the process of synthesis (For another account of this process, see Doyle [[39], p331]). Every sentence had at least one code applied, and most were categorised using several codes (e.g. 'children prefer fruit to vegetables' or 'why eat healthily?'). Before completing this stage of the synthesis, we also examined all the text which had a given code applied to check consistency of interpretation and to see whether additional levels of coding were needed. (In grounded theory this is termed 'axial' coding; see Fisher [55] for further discussion of the application of axial coding in research synthesis.) This process created a total of 36 initial codes. For example, some of the text we coded as "bad food = nice, good food = awful" from one study [56] were:

*'All the things that are bad for you are nice and all the things that are good for you are awful.' *(Boys, year 6) [[56], p74]

*'All adverts for healthy stuff go on about healthy things. The adverts for unhealthy things tell you how nice they taste.' *[[56], p75]

*Some children reported throwing away foods they knew had been put in because they were 'good for you' and only ate the crisps and chocolate*. [[56], p75]

**Figure 2:**
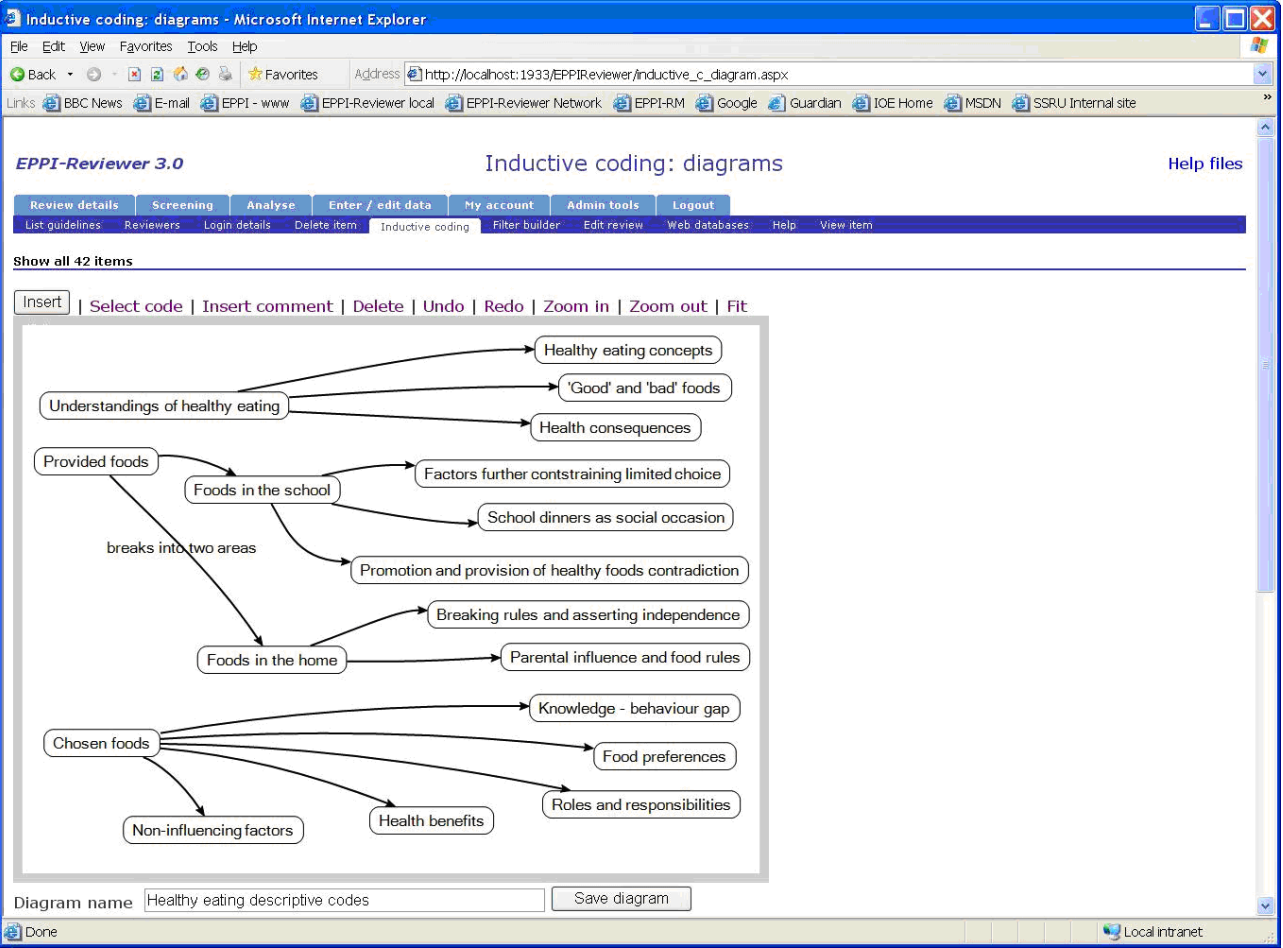
relationships between descriptive themes.

Reviewers looked for similarities and differences between the codes in order to start grouping them into a hierarchical tree structure. New codes were created to capture the meaning of groups of initial codes. This process resulted in a tree structure with several layers to organize a total of 12 descriptive themes (Figure 2). For example, the first layer divided the 12 themes into whether they were concerned with children's understandings of healthy eating or influences on children's food choice. The above example, about children's preferences for food, was placed in both areas, since the findings related both to children's reactions to the foods they were given, and to how they behaved when given the choice over what foods they might eat. A draft summary of the findings across the studies organized by the 12 descriptive themes was then written by one of the review authors. Two other review authors commented on this draft and a final version was agreed.

#### Stage three: generating analytical themes

Up until this point, we had produced a synthesis which kept very close to the original findings of the included studies. The findings of each study had been combined into a whole via a listing of themes which described children's perspectives on healthy eating. However, we did not yet have a synthesis product that addressed directly the concerns of our review – regarding how to promote healthy eating, in particular fruit and vegetable intake, amongst children. Neither had we 'gone beyond' the findings of the primary studies and generated additional concepts, understandings or hypotheses. As noted earlier, the idea or step of 'going beyond' the content of the original studies has been identified by some as the defining characteristic of synthesis [32,14].

This stage of a qualitative synthesis is the most difficult to describe and is, potentially, the most controversial, since it is dependent on the judgement and insights of the reviewers. The equivalent stage in meta-ethnography is the development of 'third order interpretations' which go beyond the content of original studies [32,11]. In our example, the step of 'going beyond' the content of the original studies was achieved by using the descriptive themes that emerged from our inductive analysis of study findings to answer the review questions we had temporarily put to one side. Reviewers *inferred *barriers and facilitators from the views children were expressing about healthy eating or food in general, captured by the descriptive themes, and then considered the implications of children's views for intervention development. Each reviewer first did this independently and then as a group. Through this discussion more abstract or analytical themes began to emerge. The barriers and facilitators and implications for intervention development were examined again in light of these themes and changes made as necessary. This cyclical process was repeated until the new themes were sufficiently abstract to describe and/or explain all of our initial descriptive themes, our inferred barriers and facilitators and implications for intervention development.

For example, five of the 12 descriptive themes concerned the influences on children's choice of foods (food preferences, perceptions of health benefits, knowledge behaviour gap, roles and responsibilities, non-influencing factors). From these, reviewers inferred several barriers and implications for intervention development. Children identified readily that taste was the major concern for them when selecting food and that health was either a secondary factor or, in some cases, a reason for rejecting food. Children also felt that buying healthy food was not a legitimate use of their pocket money, which they would use to buy sweets that could be enjoyed with friends. These perspectives indicated to us that branding fruit and vegetables as a 'tasty' rather than 'healthy' might be more effective in increasing consumption. As one child noted astutely, *'All adverts for healthy stuff go on about healthy things. The adverts for unhealthy things tell you how nice they taste.' *[[56], p75]. We captured this line of argument in the *analytical theme *entitled 'Children do not see it as their role to be interested in health'. Altogether, this process resulted in the generation of six *analytical themes *which were associated with ten recommendations for interventions.

## Results

Six main issues emerged from the studies of children's views: (1) children do not see it as their role to be interested in health; (2) children do not see messages about future health as personally relevant or credible; (3) fruit, vegetables and confectionery have very different meanings for children; (4) children actively seek ways to exercise their own choices with regard to food; (5) children value eating as a social occasion; and (6) children see the contradiction between what is promoted in theory and what adults provide in practice. The review found that most interventions were based in school (though frequently with parental involvement) and often combined learning about the health benefits of fruit and vegetables with 'hands-on' experience in the form of food preparation and taste-testing. Interventions targeted at people with particular risk factors worked better than others, and multi-component interventions that combined the promotion of physical activity with healthy eating did not work as well as those that only concentrated on healthy eating. The studies of children's views suggested that fruit and vegetables should be treated in different ways in interventions, and that messages should not focus on health warnings. Interventions that were in line with these suggestions tended to be more effective than those which were not.

## Discussion

### Context and rigour in thematic synthesis

The process of translation, through the development of descriptive and analytical themes, can be carried out in a rigorous way that facilitates transparency of reporting. Since we aim to produce a synthesis that both generates *'abstract and formal theories' *that are nevertheless *'empirically faithful to the cases from which they were developed' *[[53], p1371], we see the explicit recording of the development of themes as being central to the method. The use of software as described can facilitate this by allowing reviewers to examine the contribution made to their findings by individual studies, groups of studies, or sub-populations within studies.

Some may argue against the synthesis of qualitative research on the grounds that the findings of individual studies are de-contextualised and that concepts identified in one setting are not applicable to others [32]. However, the act of synthesis could be viewed as similar to the role of a research user when reading a piece of qualitative research and deciding how useful it is to their own situation. In the case of synthesis, reviewers translate themes and concepts from one situation to another and can always be checking that each transfer is valid and whether there are any reasons that understandings gained in one context might not be transferred to another. We attempted to preserve context by providing structured summaries of each study detailing aims, methods and methodological quality, and setting and sample. This meant that readers of our review were able to judge for themselves whether or not the contexts of the studies the review contained were similar to their own. In the synthesis we also checked whether the emerging findings really were transferable across different study contexts. For example, we tried throughout the synthesis to distinguish between participants (e.g. boys and girls) where the primary research had made an appropriate distinction. We then looked to see whether some of our synthesis findings could be attributed to a particular group of children or setting. In the event, we did not find any themes that belonged to a specific group, but another outcome of this process was a realisation that the contextual information given in the reports of studies was very restricted indeed. It was therefore difficult to make the best use of context in our synthesis.

In checking that we were not translating concepts into situations where they did not belong, we were following a principle that others have followed when using synthesis methods to build grounded formal theory: that of grounding a text in the context in which it was constructed. As Margaret Kearney has noted *"the conditions under which data were collected, analysis was done, findings were found, and products were written for each contributing report should be taken into consideration in developing a more generalized and abstract model" *[[14], p1353]. Britten *et al*. [32] suggest that it may be important to make a deliberate attempt to include studies conducted across diverse settings to achieve the higher level of abstraction that is aimed for in a meta-ethnography.

### Study quality and sensitivity analyses

We assessed the 'quality' of our studies with regard to the degree to which they represented the views of their participants. In doing this, we were locating the concept of 'quality' within the context of the purpose of our review – children's views – and not necessarily the context of the primary studies themselves. Our 'hierarchy of evidence', therefore, did not prioritise the research design of studies but emphasised the ability of the studies to answer our review question. A traditional systematic review of controlled trials would contain a quality assessment stage, the purpose of which is to exclude studies that do not provide a reliable answer to the review question. However, given that there were no accepted – or empirically tested – methods for excluding qualitative studies from syntheses on the basis of their quality [57,12,58], we included all studies regardless of their quality.

Nevertheless, our studies did differ according to the quality criteria they were assessed against and it was important that we considered this in some way. In systematic reviews of trials, 'sensitivity analyses' – analyses which test the effect on the synthesis of including and excluding findings from studies of differing quality – are often carried out. Dixon-Woods *et al*. [12] suggest that assessing the feasibility and worth of conducting sensitivity analyses within syntheses of qualitative research should be an important focus of synthesis methods work. After our thematic synthesis was complete, we examined the relative contributions of studies to our final analytic themes and recommendations for interventions. We found that the poorer quality studies contributed comparatively little to the synthesis and did not contain many unique themes; the better studies, on the other hand, appeared to have more developed analyses and contributed most to the synthesis.

## Conclusion

This paper has discussed the rationale for reviewing and synthesising qualitative research in a systematic way and has outlined one specific approach for doing this: thematic synthesis. While it is not the only method which might be used – and we have discussed some of the other options available – we present it here as a tested technique that has worked in the systematic reviews in which it has been employed.

We have observed that one of the key tasks in the synthesis of qualitative research is the translation of concepts between studies. While the activity of translating concepts is usually undertaken in the few syntheses of qualitative research that exist, there are few examples that specify the detail of how this translation is actually carried out. The example above shows how we achieved the translation of concepts across studies through the use of line-by-line coding, the organisation of these codes into descriptive themes, and the generation of analytical themes through the application of a higher level theoretical framework. This paper therefore also demonstrates how the methods and process of a thematic synthesis can be written up in a transparent way.

This paper goes some way to addressing concerns regarding the use of thematic analysis in research synthesis raised by Dixon-Woods and colleagues who argue that the approach can lack transparency due to a failure to distinguish between 'data-driven' or 'theory-driven' approaches. Moreover they suggest that, *"if thematic analysis is limited to summarising themes reported in primary studies, it offers little by way of theoretical structure within which to develop higher order thematic categories..." *[[35], p47]. Part of the problem, they observe, is that the precise methods of thematic synthesis are unclear. Our approach contains a clear separation between the 'data-driven' descriptive themes and the 'theory-driven' analytical themes and demonstrates how the review questions provided a theoretical structure within which it became possible to develop higher order thematic categories.

The theme of 'going beyond' the content of the primary studies was discussed earlier. Citing Strike and Posner [59], Campbell *et al*. [[11], p672] also suggest that synthesis *"involves some degree of conceptual innovation, or employment of concepts not found in the characterisation of the parts and a means of creating the whole"*. This was certainly true of the example given in this paper. We used a series of questions, derived from the main topic of our review, to focus an examination of our descriptive themes and we do not find our recommendations for interventions contained in the findings of the primary studies: these were new propositions generated by the reviewers in the light of the synthesis. The method also demonstrates that it is possible to synthesise without conceptual innovation. The initial synthesis, involving the translation of concepts between studies, was necessary in order for conceptual innovation to begin. One could argue that the conceptual innovation, in this case, was only necessary because the primary studies did not address our review question directly. In situations in which the primary studies are concerned directly with the review question, it may not be necessary to go beyond the contents of the original studies in order to produce a satisfactory synthesis (see, for example, Marston and King, [60]). Conceptually, our *analytical themes *are similar to the ultimate product of meta-ethnographies: *third order interpretations *[11], since both are explicit mechanisms for going beyond the content of the primary studies and presenting this in a transparent way. The main difference between them lies in their purposes. *Third order interpretations *bring together the implications of translating studies into one another in their own terms, whereas *analytical themes *are the result of interrogating a descriptive synthesis by placing it within an external theoretical framework (our review question and sub-questions). It may be, therefore, that *analytical themes *are more appropriate when a specific review question is being addressed (as often occurs when informing policy and practice), and *third order interpretations *should be used when a body of literature is being explored in and of itself, with broader, or emergent, review questions.

This paper is a contribution to the current developmental work taking place in understanding how best to bring together the findings of qualitative research to inform policy and practice. It is by no means the only method on offer but, by drawing on methods and principles from qualitative primary research, it benefits from the years of methodological development that underpins the research it seeks to synthesise.

## Competing interests

The authors declare that they have no competing interests.

## Authors' contributions

Both authors contributed equally to the paper and read and approved the final manuscript.

## Pre-publication history

The pre-publication history for this paper can be accessed here:


